# The effect of repeated full immersion simulation training in ureterorenoscopy on mental workload of novice operators

**DOI:** 10.1186/s12909-019-1752-2

**Published:** 2019-08-22

**Authors:** Takashige Abe, Faizan Dar, Passakorn Amnattrakul, Abdullatif Aydin, Nicholas Raison, Nobuo Shinohara, Muhammad Shamim Khan, Kamran Ahmed, Prokar Dasgupta

**Affiliations:** 1 MRC Centre for Transplantation, Guy’s Hospital, King’s College London, England, SE1 1UL UK; 2 Department of Urology, Hokkaido University Graduate School of Medicine, North-15, West-7, North Ward, Sapporo, 060-8638 Japan

**Keywords:** Ureterorenoscopy, Simulation training, Mental workload, NASA-TLX

## Abstract

**Background:**

Difficult surgical procedures may result in a higher mental workload, leading to increased fatigue and subsequent errors. This study was aimed to investigate the effect of repeated simulation training in ureterorenoscopy in a high-fidelity setting on the performance and mental workload of novice operators.

**Methods:**

Medical students voluntarily participated in the present simulation study. After a didactic and video-based lecture, they underwent simulation training involving a renal stone case, including a rigid cystoscope component (task 1, performing a WHO checklist, assembling a scope, and insertion of a guide-wire and an access sheath after examining the bladder) and a flexible ureterorenoscope component (task 2, retrieving a stone located in the upper calyx using a basket after inspecting the upper, middle, and lower calyx). Training was performed in a mock operating theater. Technical skills were assessed by one author (an experienced urologist) onsite using an Objective Structured Assessment of Technical Skills (OSATS) score at each training session. The mental workload was subjectively evaluated by the National Aeronautics and Space Administration Task Load Index (NASA-TLX) questionnaire after each training session.

**Results:**

Seventeen students completed a minimum of 6 training sessions (male: female = 10: 7, median age of 22) over a median of 21 days (range, 10–32). In both tasks 1 and 2, the OSATS score improved over the 6 sessions with evidence of plateauing (MANOVA model, task 1: *p* < 0.0001, task 2: *p* < 0.0001). In contrast, the NASA-TLX score persistently decreased without plateauing (task 1: *p* = 0.0005, task 2: *p* = 0.0028).

**Conclusions:**

Under repeated simulation training in ureterorenoscopy in a high-fidelity setting, participants showed a continual decrease of the mental workload, while the improvement of technical skills reached a plateau over the 6 sessions. Our study showed the important benefit of simulation training to reduce the mental workload by repeated scenario training before actual clinical practice.

**Electronic supplementary material:**

The online version of this article (10.1186/s12909-019-1752-2) contains supplementary material, which is available to authorized users.

## Background

With the widespread use of minimally invasive surgeries such as endoscopy and laparoscopy, limitations to training as a result of working-hour regulations, and the ethical consideration of patient safety, simulation training is now considered an important part of surgical education. In the urological field, ureterorenoscopy is one of the most frequently performed minimally invasive procedures to manage urinary stones or upper urinary tract cancer. The lack of haptic feedback, limited visual field, and sheer quantity of instruments such as rigid/flexible scopes, guide-wires, access sheaths, and baskets that the surgeon needs to be familiar with, means ureterorenoscopy is one of the most technically difficult and stressful procedures especially for novice operators. The associated stress may increase fatigue and have a negative impact on surgical performance.

Various studies of simulation-based ureterorenoscopy training have been reported, with most focusing on the validation of an individual training model or technical skill improvement [1–6]. There is a paucity of literature analyzing the impact of repeated simulation training on changes in the mental workload of novice operators. However, such studies could offer valuable information to promote patient safety and develop an effective educational training curriculum. The aim of the present study was to investigate the effect of repeated simulation-based ureterorenoscopy training in a high-fidelity setting on the mental workload and the skill achievements of novice operators.

## Methods

### Participants

Nineteen medical students, with no prior ureterorenoscopy training, voluntary participated in the present simulation training and provided written informed consent. After obtaining background information including the age, sex, clinical experience (medical school year), dominant hand, and experience of urological endoscopy, all participants initially received video-based didactic training on the basics of cystoscopy and ureterorenoscopy, stone treatment, devices such as guide-wires, access sheaths, baskets, and on how to perform the following two tasks. After this training, each participant engaged in the first training session (1st session). Subsequently, participants underwent a minimum of five additional training sessions, with an inter-session interval of at least 5 h.

### Training task

During the training sessions, the case scenario described “a 73-year-old male without any significant past medical history who is about to undergo the removal of a stone located in the right renal upper calyx”. Table 1 shows each step the participants were required to perform. Task 1 involved training in the use of a rigid cystoscope and preparation for stone removal using a flexible ureterorenoscopy. Task 2 involved training in flexible ureterorenoscopy and stone removal with a basket. After completing the WHO checklist and assembling their devices, participants were required to perform thorough bladder mucosa observation using a rigid cystoscopy (22.5 Fr, Olympus, Japan), and place a guide-wire (PTFE guide-wire, 0.035, 150 cm, Olympus, Japan) and an access sheath (UroPass®, 12/14 Fr, 38 cm, Olympus, Japan) into the right renal orifice. They were then required to perform a systemic examination of the right renal calices using a flexible ureterorenoscopy (DUR-8®, Gyrus ACMI, USA) and extract the stone located in the upper renal calyx using a basket (Ultra-Catch NT®, 1.8 Fr, 115 cm, Olympus, Japan).

**Table 1:** Summary of the required steps in each task

Task 1	
1. Go though the WHO checklist.	
2. Assemble the rigid cystoscope.	
3. Connect the cables to the light-source devices.	
4. Turn the light-source devices on.	
5. Orient the camera to the 12 o’clock position and adjust the focus.	
6. Perform white balance.	
7. Utilize lubricant jelly (cystoscope)	
8. Observe the bladder mucosa.	
9. Identify both ureteral orifices.	
10. Utilize water as a lubricant (guide-wire)	
11. Insert a guide-wire into the right renal unit.	
12. Utilize water as a lubricant (access sheath)	
13. Insert an access sheath into the right renal unit.	
Task 2	
1. Connect the cables to the flexible ureteroscope.	
2. Orient the camera to the 12 o’clock position and adjust the focus.	
3. Perform white balance.	
4. Observe the upper calyx.	
5. Observe the middle calyx.	
6. Observe the lower calyx.	
7. Retract the scope inside the access sheath before inserting the basket.	
8. Insert the basket and catch the stone with it (two-minute time limit.)	

**Fig. 1:**
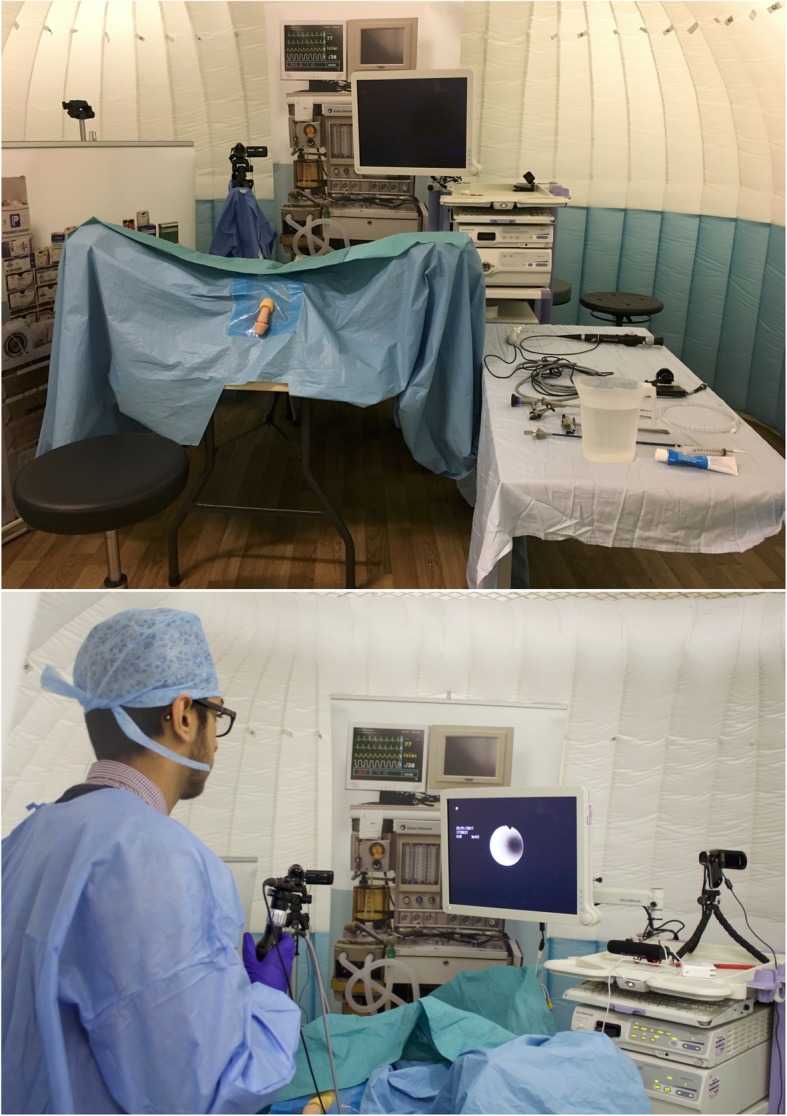
View of ureteroscopy training in a virtual theater

Training was performed in a mock operating theater, “Igloo” (Imperial College London, UK), a portable inflatable simulated operating room, with the two collaborators in the roles of an anesthetist (TA) or a scrub nurse (FD) (Fig. 1). The Uro-Scopic Trainer® (Limbs and Thighs Ltd., Bristol, UK) was used in task 1, and the Scopic Trainer® (Mediskills Ltd., Edinburgh, UK) in task 2. Before task 2, the Uro-Scopic Trainer® was replaced with the Scopic Trainer® with an access sheath in situ and a stone fragment in the upper renal calyx. During the session, participants were expected to perform each procedure independently, although one of the authors (TA, an experienced urologist) gave appropriate guidance according to the situation during the training, and conducted debriefing after each training session. The scrub nurse (FD) acted as a passive surgical assistant, performing tasks such as holding the guide-wire or operating the basket according to participants’ instructions. A maximum of 30 min was allowed for each task, and performances including both endoscopic and external views were video-recorded for subsequent analyses.

### Outcome measures

**Fig. 2:**
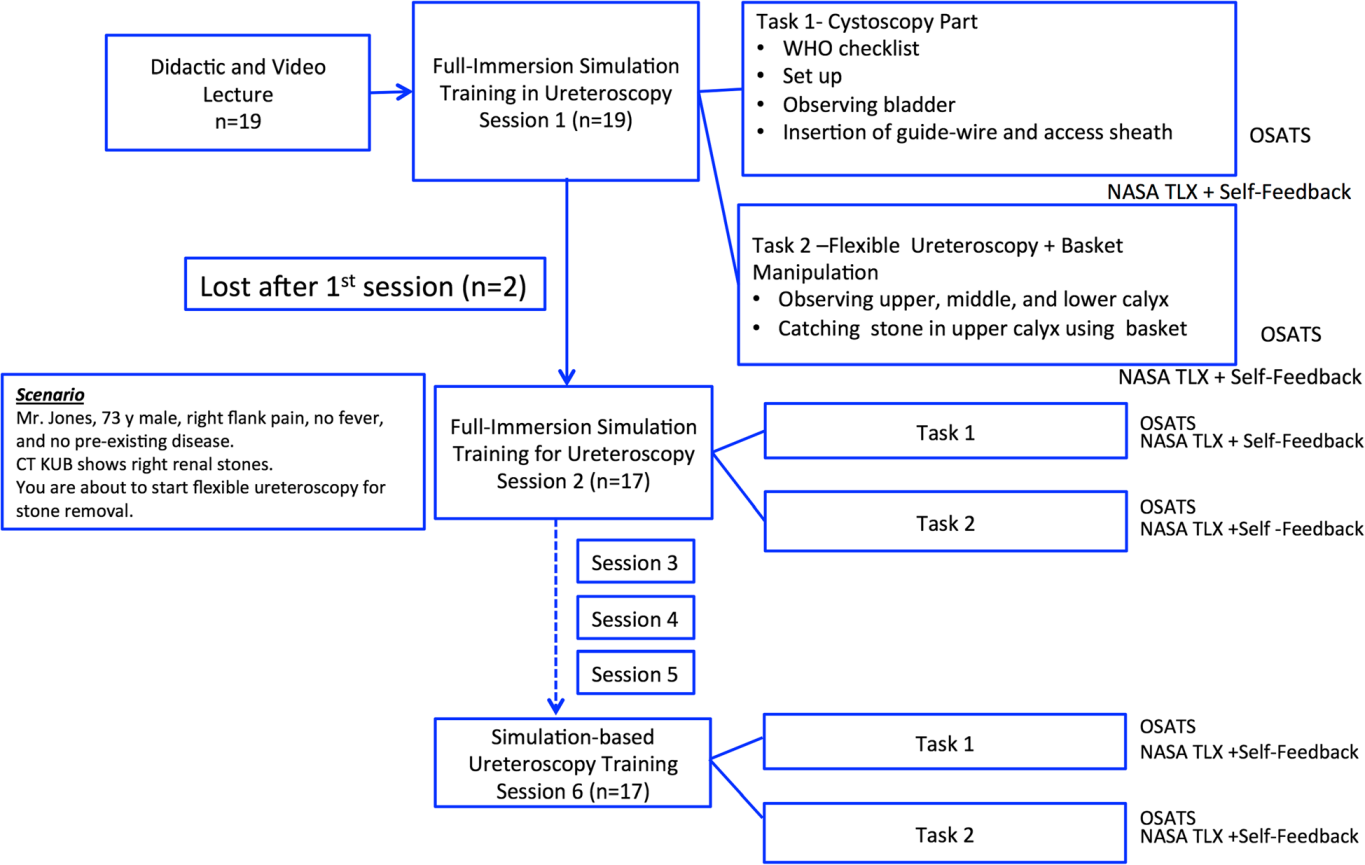
Flow of the present study

The time taken to complete each task was recorded. A procedural checklist, described in Table 1, was used to assess performance by both authors (TA and FD). The checklist score was calculated by awarding one point for each step performed correctly and independently. There was a total score of 13 points for task 1, and 8 points for task 2. Technical skills were assessed by one of the authors (TA) onsite using the Objective Structured Assessment of Technical Skills (OSATS) score [7]. Following the completion of each task, the mental workload was subjectively evaluated by each participant. The National Aeronautics and Space Administration Task Load Index (NASA-TLX), the most extensively utilized subjective questionnaire for mental workload assessment, was used [8]. The NASA-TLX includes six subscales: mental, physical, and temporal task demands, effort, frustration, and perceived performance, and the questionnaire uses a 20-point visual analogue scale to measure the mental workload based on the 6 above-mentioned subscales. Participants also completed a self-assessment-based questionnaire on difficulty and confidence after each training session (Additional file 1: Table S1). Figure 2 shows the flow of the present study. To evaluate assessment reliability, 30 videos were selected (1st, 3rd, and 6th session movies of 10 random participants), and assessed by a blinded expert ureteroscopist (PA) using the OSATS score.

### Statistical methods

Differences in the NASA-TLX score between tasks 1 and 2 were assessed using a paired t-test. A MANOVA model was used to analyze differences in task times and scores over training sessions. Pearson correlation coefficients were assessed between the onsite and blinded OSATS scores. All statistical analyses were performed using JMP® Pro12.01 (SAS). *P*-values < 0.05 were considered significant.

## Results

After the 1st training session, two students withdrew, and the remaining seventeen students who had completed a minimum of 6 training sessions were included in the present analyses. Table 2 shows a summary of participants’ characteristics. The median age was 22 years (range, 18–35), and ten were male. All participants were right-handed and five had experience of observing urological endoscopic procedures.

**Table 2:** Summary of participants’ characteristics

	Total, *n* = 17
Age, years	Median 22 (range, 18–35)
Sex	M/F = 10/7
Medical school year	Median 3 (range, 1–5)
Dominant hand	Right = 17
Observational experience of urological endoscopy	Yes/No = 5/12
Training session completed, times	Median 6 (range, 6–9)
Time to complete 6 training sessions, days	Median 21 (range, 10–32)

**Fig. 3:**
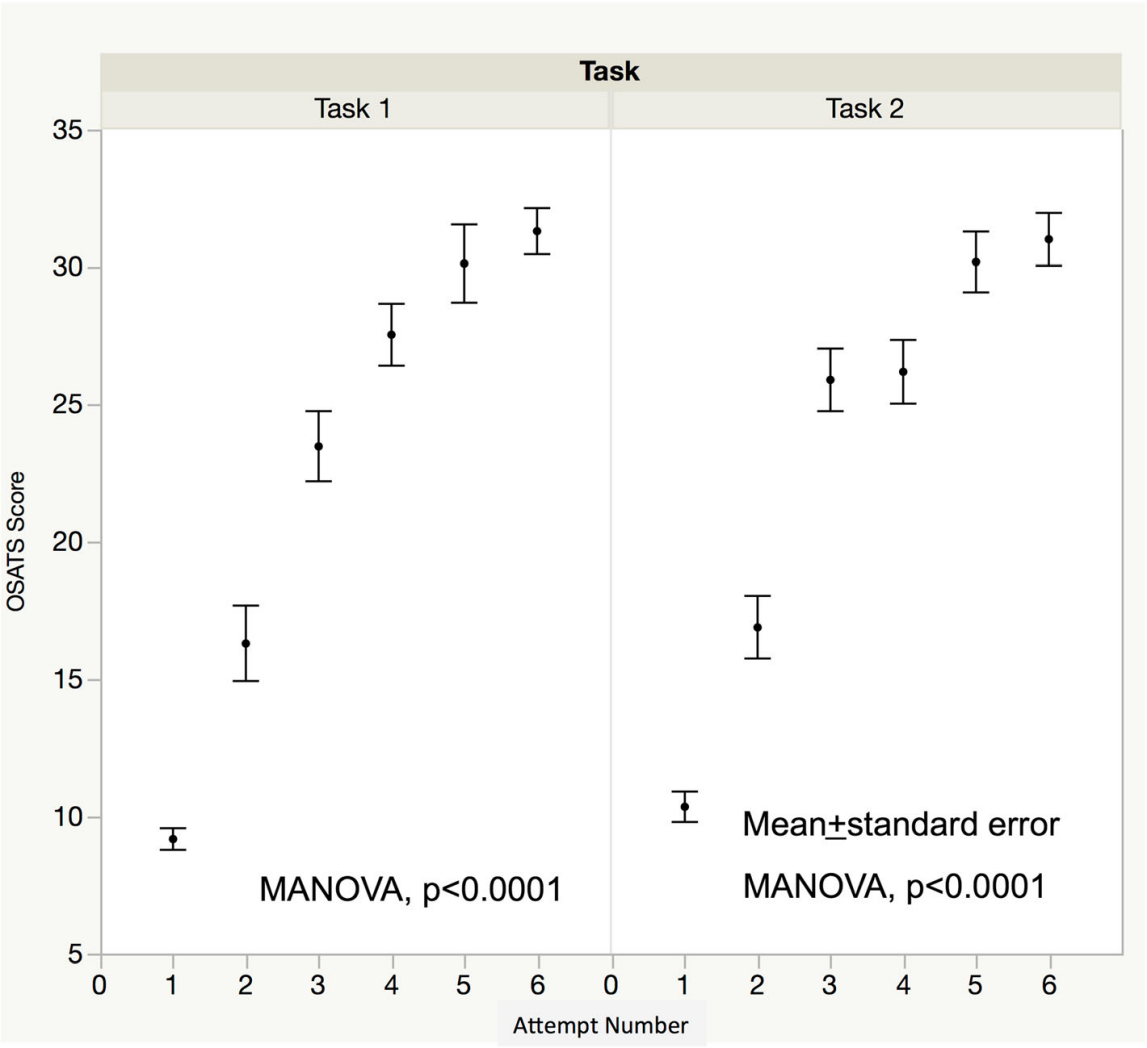
OSATS scores over the 6 sessions. The OSATS scores significantly improved over the 6 sessions for both tasks

**Fig. 4:**
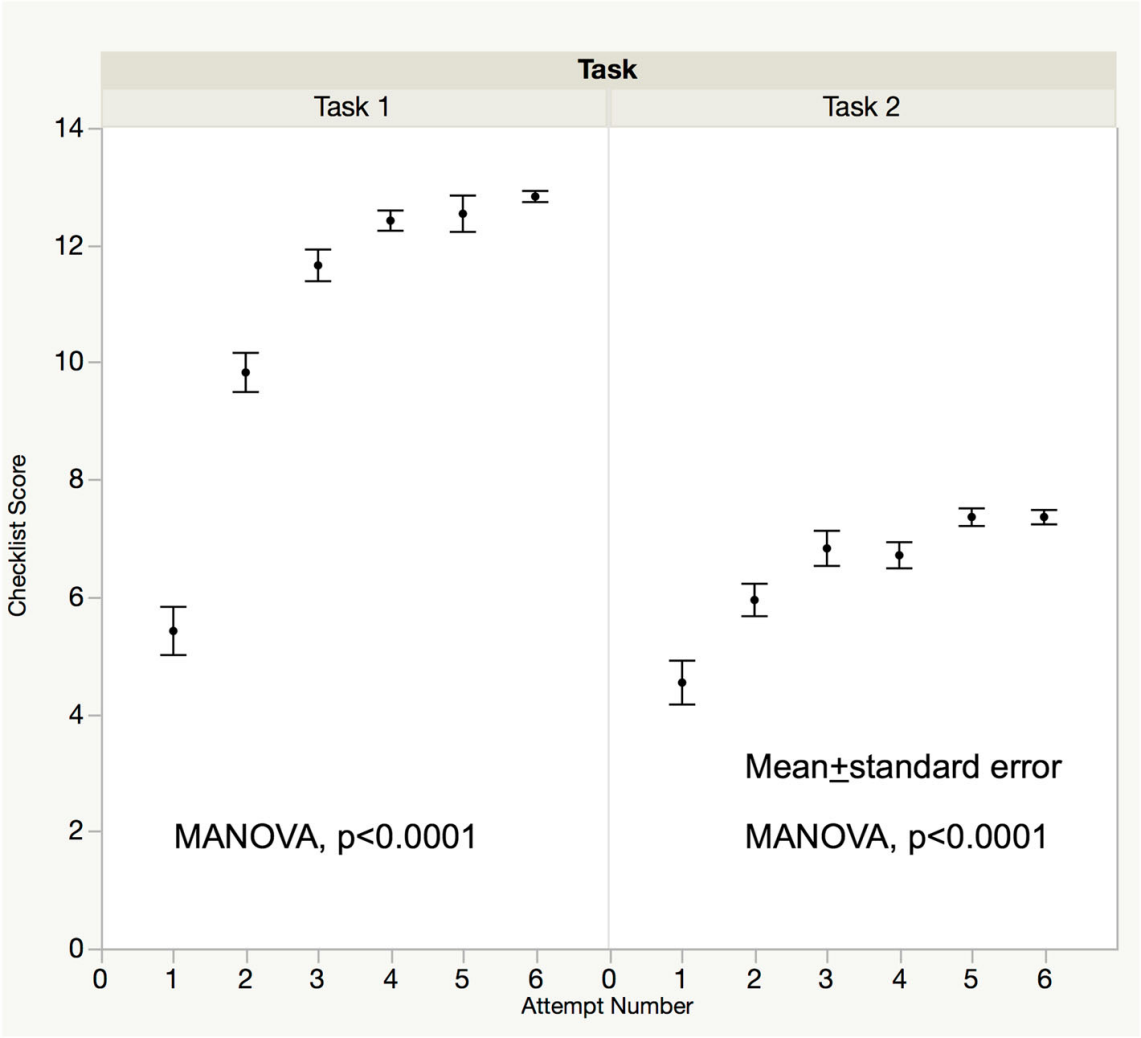
Checklist scores over the 6 sessions. Checklist scores also improved over the sessions

**Fig. 5:**
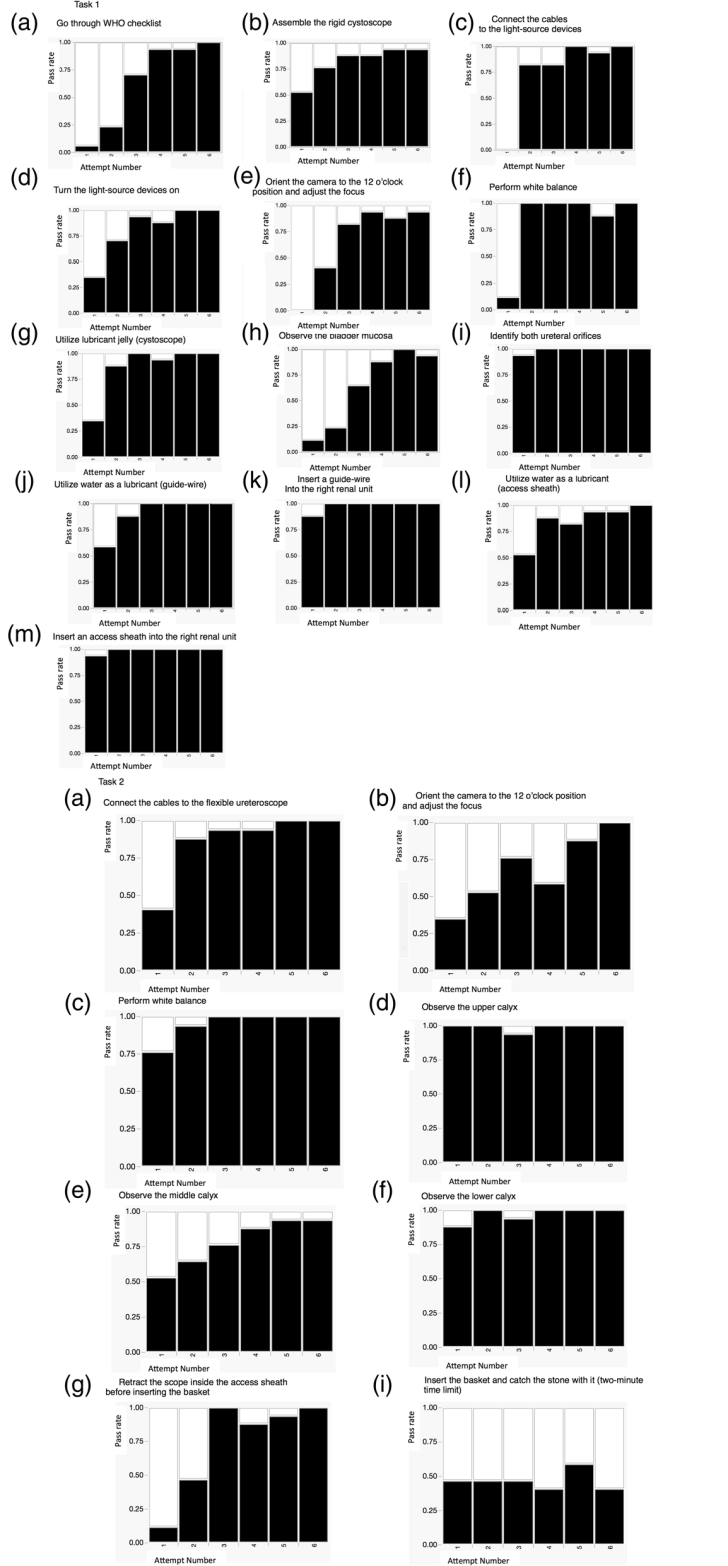
Pass rate of each surgical step over the sessions. All the steps except for “Insert the basket and catch the stone with it (two-minute time limit)” were accomplished over the training sessions

Table 3 shows the mean time ± standard deviation to complete each task in the 1st, 3rd, and 6th sessions. The total operative time decreased over the course of training. However, when the total time for task 2 was divided into two parts (before and after initiation of the basket procedure) there was no significant improvement in the time needed to catch the stone in the basket during the 6 sessions. Figure 3 shows OSATS scores over the 6 sessions. Scores improved over the 6 sessions in both tasks with evidence of plateauing. Inter-rater reliability of the OSATS scores was high (Additional file 2: Figure S1 r = 0.8776, *p* < 0.0001). Checklist scores also improved over the sessions with similar plateauing in score progression (Fig. 4). All the steps except “Insert the basket and catch the stone with it (two-minute time limit)” were accomplished over the training sessions, as shown in Fig. 5.

**Table 3:** Summary of time to complete each task in 1st, 3rd, and 6th sessions

	1st, mean ± SD	3rd, mean ± SD	6th, mean ± SD	*p* -value, MANOVA
Task 1, seconds	1077 ± 207	546 ± 99	394 ± 56	< 0.0001
Task 2, total, seconds	830 ± 252	639 ± 417	442 ± 163	0.0062
Before basket, seconds	591 ± 125	385 ± 172	229 ± 30	< 0.0001
After basket, seconds	239 ± 212	253 ± 280	213 ± 171	0.7622

*SD* standard deviation

**Fig. 6:**
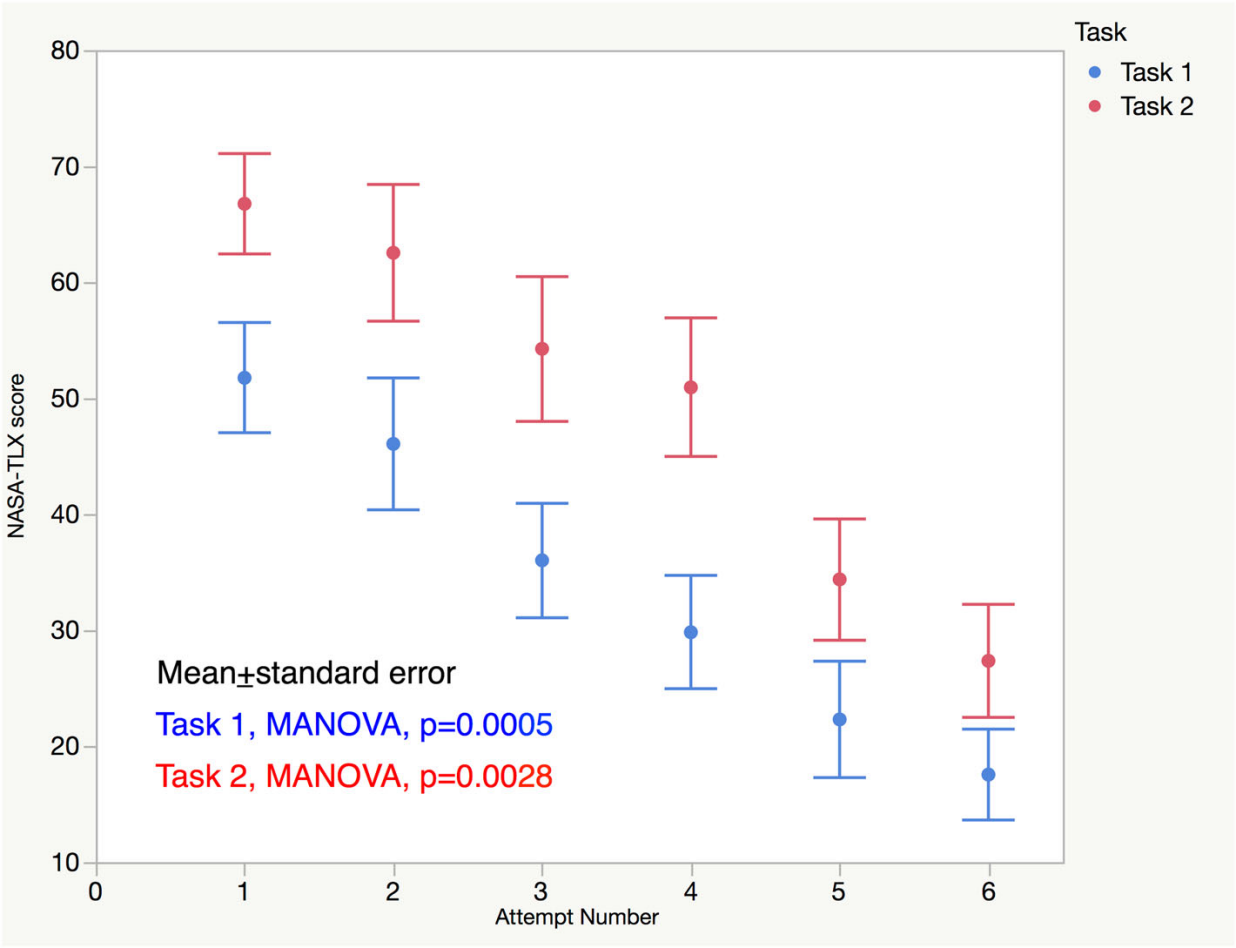
NASA-TLX scores over the training sessions. The mental workload was significantly higher in task 2 than task 1 in every training session (paired t-test, *p* < 0.05). During the training sessions, there was a steady decline in the mental workload in both tasks

Fig. 6 shows NASA-TLX scores during the training sessions. The mental workload was significantly higher in task 2 than in task 1 in every training session (paired t-test, *p* < 0.05). During the training sessions, there was a continual improvement in the mental workload in both tasks without plateauing (MANOVA model, task 1: *p* = 0.0005, task 2: *p* = 0.0028). The self-assessment scores for difficulty and confidence were consistent with the results of NASA-TLX. Participants showed improved self-confidence after repeated training (Additional file 3: Figure S2).

## Discussion

Regarding the effect of simulation training on the operator’s workload, Hu et al. observed a decreased mental workload in three basic laparoscopic tasks: ring transfer, precision cutting, and intracorporeal suturing, based on the Fundamentals of Laparosocpic Surgery (FLS) curriculum [9], after simulation training. Following one-hour training, medical students demonstrated improved technical performance, and significant reductions in NASA-TLX scores [10]. Yurko et al., in their study including 28 novices, also observed a reduced mental workload in laparoscopic suturing and knot tying after subjects had achieved a previously published expert-level performance score [11]. In the present study, the 17 participants underwent simulation training in cystoscopy and subsequent ureterorenoscopy in a high-fidelity setting multiple times. Our study demonstrates the effectiveness of structured, high-fidelity scenario training with appropriate guidance and debriefing from expert urologic surgeons. Repeated training was beneficial for both technical skill acquisition and reducing the mental workload.

In the present study, we prepared a simple clinical scenario of stone treatment, and participants were required to complete the predetermined surgical steps independently. As shown in Fig. 5, all the steps except for “Insert the basket and catch the stone with it (two-minute time limit)” were accomplished over the training sessions. We consider that dividing a training task into required routine steps and assessing their accomplishments over training sessions is helpful to build an effective training program. Based on our observation that OSATS scores reached a plateau in the 5th and 6th sessions, we consider that five repeats of scenario-training of stone treatment after the introduction session would be sufficient to learn the core tasks of stone surgery regarding technical aspects, and training in the basket part could be conducted separately according to each trainee’s level of skill achievement. However, in terms of the mental workload, NASA-TLX scores steadily decreased over the sessions, without reaching a plateau. This study highlights that even though technical performance may plateau during simulation training, reductions in the mental workload continue to be realized. This both emphasizes the benefits of repeated, structured simulation training and highlights the importance of simulation even when participants and trainers may feel a satisfactory technical proficiency has been achieved.

The main limitation of the present study is that, whilst all efforts were made to create a realistic, high-fidelity environment, it is reasonable to consider that some of the simulation training effect will be lost when participants are confronted their initial cases in the operating theater. The lack of a real clinical section wherein we can confirm the transferability of the simulation training effect to real clinical practice is a flaw of this study, because the present subjects were medical students from different years and it was difficult to perform such a real clinical section. Furthermore, in real clinical practice, surgeons have to cope with multiple distractions simultaneously, such as staff entering the operating theater, case-irrelevant conversations, telephone calls, and equipment-related problems. In terms of operating room distractions, Wheelock et al. reported an interesting observational study. They observed ninety general surgery cases in real time and assessed them using 4 validated tools: OR Distraction Assessment Form, the Observational Teamwork Assessment for Surgery tool, NASA-TLX, and short form of the State Trait Anxiety Inventory. They observed that the most prevalent distractions were those caused by external staff, followed by case-irrelevant conversations. Case-irrelevant conversations were associated with poor team performance. Equipment-related distractions were correlated with higher stress levels and poorer teamwork in nurses [12]. It is likely that novice trainees are more susceptible to those distractions. Our group previously reported that after the simulation-based curriculum for the rigid ureteroscope, including the teaching of non-technical skills, the intervention cohort showed improvements both in technical and nontechnical aspects, compared with the control group [13]. In addition, Stefanidis et al. recently reported an interesting mental skill curriculum specific to surgery. Nine surgical novices completed their curriculum, and they showed significant improvements in their laparoscopic simulator-based performance and mental skills. All participants also completed the procedure using a porcine model without a significant change in their perceived stress [14]. In order to improve patient safety, we must continue efforts to develop an integrated surgical training program, including nontechnical skill training.

Another limitation is that we cannot comment on the possibility of changes in their performance outcomes or mental workload on using different scenarios such as a left renal stone during data collection, because participants performed training based on the same “right renal stone” scenario. If we invite novice trainees to participate in the present study, their scores might be different from the current observation in medical students, as novice trainees have real operative experience in the theater. Nevertheless, we believe that our study showed the potential benefit of mental workload reduction facilitated by simulation training before real clinical practice. Our study provided a valid scenario training model and researchers are able to examine influences of other potential stressors within the “mock” theater, using different scenarios. Further studies of surgical simulations are warranted to develop an integrated training program.

## Conclusions

Under repeated simulation training in ureterorenoscopy in a high-fidelity setting, participants showed a continual decrease of the mental workload, while the improvement of technical skills reached a plateau over 6 sessions. Our study showed the important benefit of simulation training to reduce the mental workload by repeated scenario training before actual clinical practice.

## Additional files

Additional file 1: **Table S1.** Feedback questionnaire (DOCX 22 kb)

Additional file 2: **Figure S1.** Correlation of the OSATS sores between the real-time (onsite) and blinded raters. A strong correlation was observed between the real-time (onsite) and blinded raters. (TIFF 26369 kb)

Additional file 3: **Figure S2.** Self-assessment scores of difficulty and confidence. Participants showed increased self-confidence after repeated training. (TIFF 26369 kb)

## Data Availability

The datasets generated and/or analyzed during the current study are available from the corresponding author on reasonable request.

## Abbreviations

NASA-TLX: National Aeronautics and Space Administration Task Load Index
OSATS: Objective Structured Assessment of Technical Skills

## References

[1] Ogan K, Jacomides L, Shulman MJ, Roehrborn CG, Cadeddu JA, Pearle MS (2004). Virtual ureteroscopy predicts ureteroscopic proficiency of medical students on a cadaver. J Urol.

[2] White MA, Dehaan AP, Stephens DD, Maes AA, Maatman TJ (2010). Validation of a high fidelity adult ureteroscopy and renoscopy simulator. J Urol.

[3] Brehmer M, Swartz R (2005). Training on bench models improves dexterity in ureteroscopy. Eur Urol.

[4] Villa L, Sener TE, Somani BK, Cloutier J, Buttice S, Marson F, Doizi S, Proietti S, Traxer O (2017). Initial content validation results of a new simulation model for flexible Ureteroscopy: the key-box. J Endourol.

[5] Watterson JD, Beiko DT, Kuan JK, Denstedt JD (2002). Randomized prospective blinded study validating acquistion of ureteroscopy skills using computer based virtual reality endourological simulator. J Urol.

[6] Wilhelm DM, Ogan K, Roehrborn CG, Cadeddu JA, Pearle MS (2002). Assessment of basic endoscopic performance using a virtual reality simulator. J Am Coll Surg.

[7] Martin JA, Regehr G, Reznick R, MacRae H, Murnaghan J, Hutchison C, Brown M (1997). Objective structured assessment of technical skill (OSATS) for surgical residents. Br J Surg.

[8] Hart SGSS, Hancock PA, Meshkati N (1987). Development of NASA-TLX: results of empirical and theoretical research. Human mental workload.

[9] Peters JH, Fried GM, Swanstrom LL, Soper NJ, Sillin LF, Schirmer B, Hoffman K, Committee SF (2004). Development and validation of a comprehensive program of education and assessment of the basic fundamentals of laparoscopic surgery. Surgery.

[10] Hu JS, Lu J, Tan WB, Lomanto D (2016). Training improves laparoscopic tasks performance and decreases operator workload. Surg Endosc.

[11] Yurko YY, Scerbo MW, Prabhu AS, Acker CE, Stefanidis D (2010). Higher mental workload is associated with poorer laparoscopic performance as measured by the NASA-TLX tool. Simul Healthc.

[12] Wheelock A, Suliman A, Wharton R, Babu ED, Hull L, Vincent C, Sevdalis N, Arora S (2015). The impact of operating room distractions on stress, workload, and teamwork. Ann Surg.

[13] Brunckhorst O, Shahid S, Aydin A, McIlhenny C, Khan S, Raza SJ, Sahai A, Brewin J, Bello F, Kneebone R, Khan MS, Dasgupta P, Ahmed K (2015). Simulation-based ureteroscopy skills training curriculum with integration of technical and non-technical skills: a randomised controlled trial. Surg Endosc.

[14] Stefanidis D, Anton NE, McRary G, Howley LD, Pimentel M, Davis C, Yurco AM, Sevdalis N, Brown C (2017). Implementation results of a novel comprehensive mental skills curriculum during simulator training. Am J Surg.

