# Supplementary material for: The effect of repeated full immersion simulation training in ureterorenoscopy on mental workload of novice operators
Source: BMC Med Educ. 2019 Aug 22;19:318. doi: 10.1186/s12909-019-1752-2 (PMC6704721; doi:10.1186/s12909-019-1752-2)
Supplement: Supplementary file 1 — Table S1. Feedback questionnaire (DOCX 22 kb) [file 12909_2019_1752_MOESM1_ESM.docx]

Additional file 1: Table 1. Feedback questionnaire

On a scale of 1-5, how **difficult** did you find the following steps during the training session?

(1 = Very Easy, 5 = Very Difficult)

Task 1:

1. Going through the WHO checklist: 1⬜ 2⬜ 3⬜ 4⬜ 5⬜
2. Assembling the rigid cystoscope: 1⬜ 2⬜ 3⬜ 4⬜ 5⬜
3. Turning on Lap Stack: 1⬜ 2⬜ 3⬜ 4⬜ 5⬜
4. Connecting the cables to Lap Stack: 1⬜ 2⬜ 3⬜ 4⬜ 5⬜
5. Orienting the camera and adjusting the focus: 1⬜ 2⬜ 3⬜ 4⬜ 5⬜
6. Performing white balance: 1⬜ 2⬜ 3⬜ 4⬜ 5⬜
7. Utilizing lubricant jelly: 1⬜ 2⬜ 3⬜ 4⬜ 5⬜
8. Observing the bladder: 1⬜ 2⬜ 3⬜ 4⬜ 5⬜
9. Identifying the relevant ureteric orifice: 1⬜ 2⬜ 3⬜ 4⬜ 5⬜
10. Utilizing water as a lubricant: 1⬜ 2⬜ 3⬜ 4⬜ 5⬜
11. Inserting a guide-wire: 1⬜ 2⬜ 3⬜ 4⬜ 5⬜
12. Utilizing water as a lubricant: 1⬜ 2⬜ 3⬜ 4⬜ 5⬜
13. Inserting an access sheath: 1⬜ 2⬜ 3⬜ 4⬜ 5⬜
14. Use of the assistant/scrub-nurse: 1⬜ 2⬜ 3⬜ 4⬜ 5⬜

Task 2:

1. Connecting the cables to the flexible Ureteroscope: 1⬜ 2⬜ 3⬜ 4⬜ 5⬜
2. Orienting the camera and adjusting the focus: 1⬜ 2⬜ 3⬜ 4⬜ 5⬜
3. Performing white balance: 1⬜ 2⬜ 3⬜ 4⬜ 5⬜
4. Utilizing lubricant jelly: 1⬜ 2⬜ 3⬜ 4⬜ 5⬜
5. Observing the upper calyx: 1⬜ 2⬜ 3⬜ 4⬜ 5⬜
6. Observing the middle calyx: 1⬜ 2⬜ 3⬜ 4⬜ 5⬜
7. Observing the lower calyx: 1⬜ 2⬜ 3⬜ 4⬜ 5⬜
8. Catching hold of the stone with the basket: 1⬜ 2⬜ 3⬜ 4⬜ 5⬜
9. Use of the assistant/scrub -nurse: 1⬜ 2⬜ 3⬜ 4⬜ 5⬜

On a scale of 1-5, how **confident** are you in completing the following steps in your next training session?

(1 = Not Confident, 5 = Very Confident)

Task 1:

1. Going through the WHO checklist: 1⬜ 2⬜ 3⬜ 4⬜ 5⬜
2. Assembling the rigid cystoscope: 1⬜ 2⬜ 3⬜ 4⬜ 5⬜
3. Turning on Lap Stack: 1⬜ 2⬜ 3⬜ 4⬜ 5⬜
4. Connecting the cables to Lap Stack: 1⬜ 2⬜ 3⬜ 4⬜ 5⬜
5. Orienting the camera and adjusting the focus: 1⬜ 2⬜ 3⬜ 4⬜ 5⬜
6. Performing white balance: 1⬜ 2⬜ 3⬜ 4⬜ 5⬜
7. Utilizing lubricant jelly: 1⬜ 2⬜ 3⬜ 4⬜ 5⬜
8. Observing the bladder: 1⬜ 2⬜ 3⬜ 4⬜ 5⬜
9. Identifying the relevant ureteric orifice: 1⬜ 2⬜ 3⬜ 4⬜ 5⬜
10. Utilizing water as a lubricant: 1⬜ 2⬜ 3⬜ 4⬜ 5⬜
11. Inserting a guide-wire: 1⬜ 2⬜ 3⬜ 4⬜ 5⬜
12. Utilizing water as a lubricant: 1⬜ 2⬜ 3⬜ 4⬜ 5⬜
13. Inserting an access sheath: 1⬜ 2⬜ 3⬜ 4⬜ 5⬜
14. Use of the assistant/scrub-nurse: 1⬜ 2⬜ 3⬜ 4⬜ 5⬜

Task 2:

1. Connecting the cables to the flexible ureteroscope: 1⬜ 2⬜ 3⬜ 4⬜ 5⬜
2. Orienting the camera and adjusting the focus: 1⬜ 2⬜ 3⬜ 4⬜ 5⬜
3. Performing white balance: 1⬜ 2⬜ 3⬜ 4⬜ 5⬜
4. Utilizing lubricant jelly: 1⬜ 2⬜ 3⬜ 4⬜ 5⬜
5. Observing the upper calyx: 1⬜ 2⬜ 3⬜ 4⬜ 5⬜
6. Observing the middle calyx: 1⬜ 2⬜ 3⬜ 4⬜ 5⬜
7. Observing the lower calyx: 1⬜ 2⬜ 3⬜ 4⬜ 5⬜
8. Catching the stone with the basket: 1⬜ 2⬜ 3⬜ 4⬜ 5⬜
9. Use of the assistant/scrub-nurse: 1⬜ 2⬜ 3⬜ 4⬜ 5⬜
