# Supplementary figures and images for: The effect of repeated full immersion simulation training in ureterorenoscopy on mental workload of novice operators
Source: BMC Med Educ. 2019 Aug 22;19:318. doi: 10.1186/s12909-019-1752-2 (PMC6704721; doi:10.1186/s12909-019-1752-2)

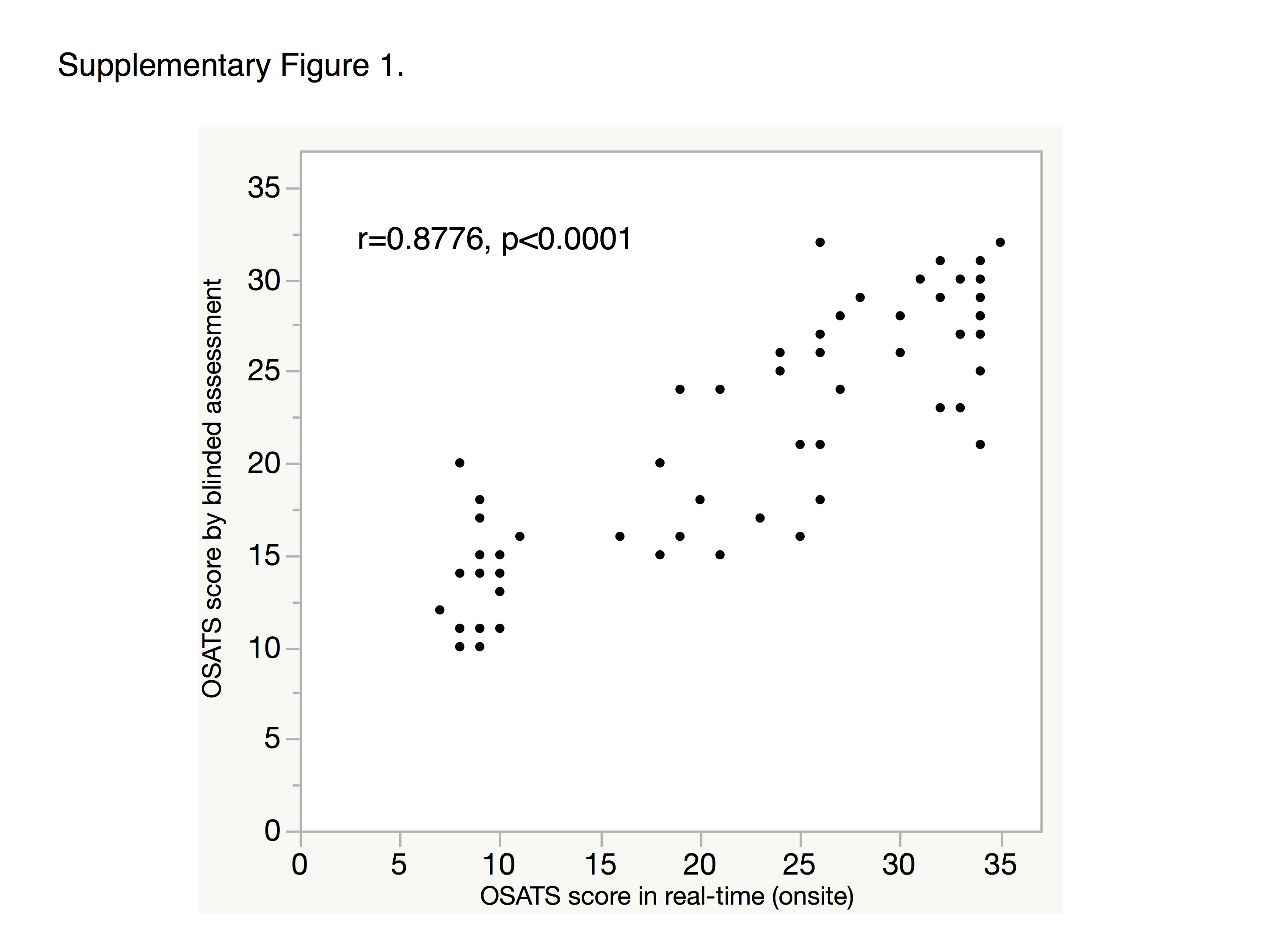

Supplement: Supplementary file 2 — Figure S1. Correlation of the OSATS sores between the real-time (onsite) and blinded raters. A strong correlation was observed between the real-time (onsite) and blinded raters. (TIFF 26369 kb) [file 12909_2019_1752_MOESM2_ESM.tiff]

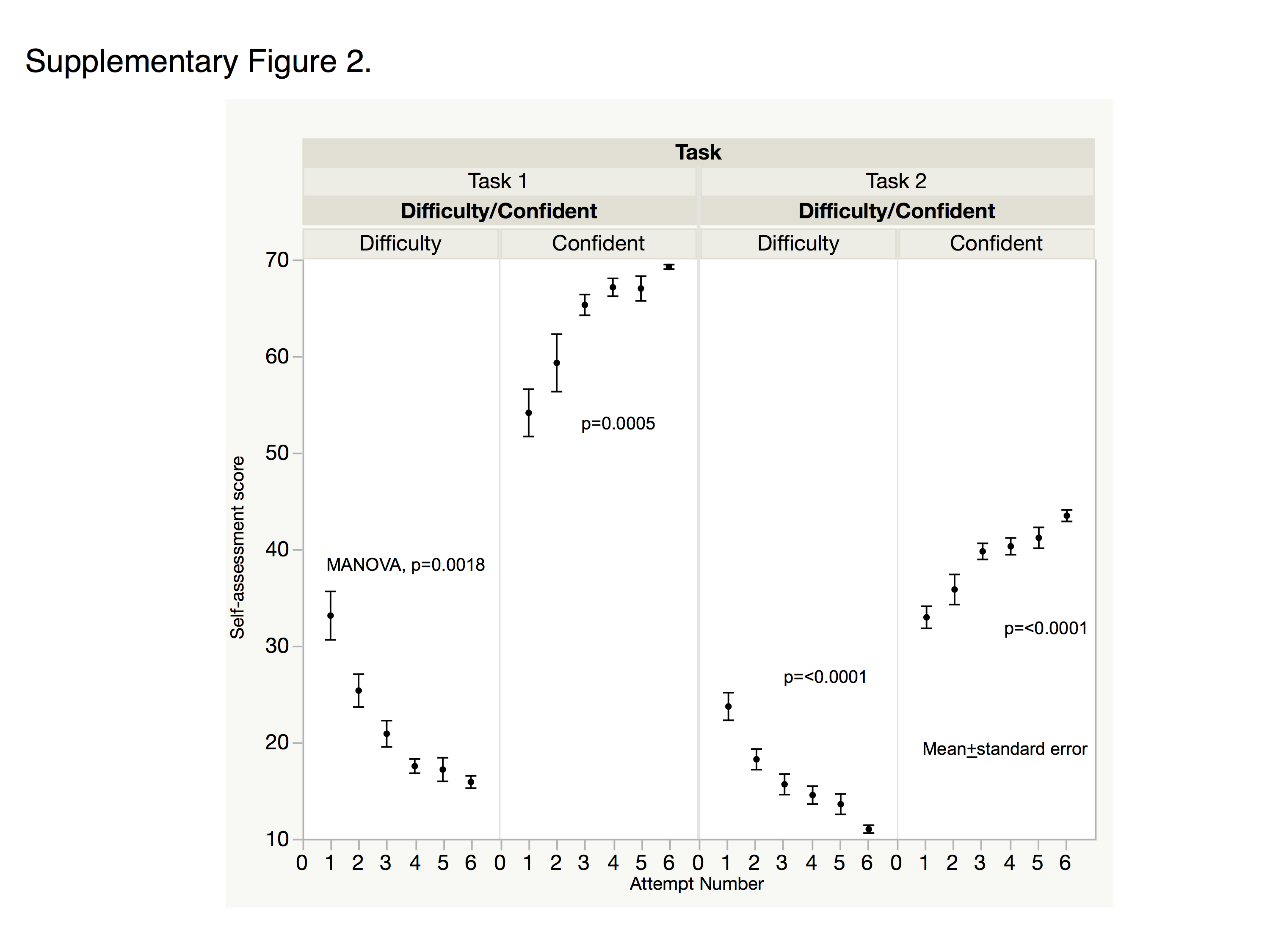

Supplement: Supplementary file 3 — Figure S2. Self-assessment scores of difficulty and confidence. Participants showed increased self-confidence after repeated training. (TIFF 26369 kb) [file 12909_2019_1752_MOESM3_ESM.tiff]
